# Against the Stream: Generalised anxiety disorder (GAD) – a redundant diagnosis

**DOI:** 10.1192/bjb.2017.12

**Published:** 2018-04

**Authors:** Peter Tyrer

**Affiliations:** 1 Imperial College London, UK

## Abstract

The diagnosis of generalised anxiety disorder is a distraction of no value. It is highly unreliable, co-occurring with many other disorders of firmer diagnostic status, and has intrinsic connections to personality dysfunction. It is argued that classification would be heartily relieved to rid itself of this unnecessary appendage and for the symptom of anxiety to remain as a descriptive term only.

**Declaration of interest:**

None.

Generalised anxiety disorder (GAD) describes an excessive and disproportionate anxiety or worry about minor matters that would not trouble most people. In addition to mental tension, consequent muscle pain and inability to relax, people with GAD almost always have bodily symptoms such as shaking, nausea, tingling sensations, a variety of gastrointestinal symptoms covering the full extremity from mouth to anus, sweating, palpitations, breathlessness and other respiratory symptoms. It is a recurrent disorder and often lasts for many years. This is an easily recognisable syndrome that is found to be one of the most common in population epidemiological surveys.

So why do I suggest that the letters GAD would better stand for godawful diagnosis? It is because all the characteristics that make a diagnosis useful in psychiatry – clear and reliable presenting symptoms, predictable outcome and treatment response – are lacking. Each of the components of the diagnosis has been systematically stripped away, leaving an ill-defined core of excessive worry only, which makes the diagnosis so grossly comorbid as to be useless. Let us take each of these elements in turn.

## Excessive and disproportionate anxiety

If there is sudden and dramatic anxiety occurring without obvious reason, particularly when accompanied by bodily symptoms such as palpitations and shortness of breath, the condition is described as panic disorder. There is no difference between the symptoms of panic disorder and GAD; only their speed of onset separates them. Panic disorder is like an overpowered car that can go from zero to 60 mph in 5 s, while GAD is like a reconditioned Morris Minor that takes at least a minute to get to 60 mph and then groans with it and about it, for years. The removal of panic makes GAD into worry only.

## Bodily symptoms

### Mid-gastrointestinal – irritable bowel syndrome

Irritable bowel syndrome is now so well known that even the general public refer to it as IBS. Most people with IBS are anxious^1^ and often have other bodily symptoms of anxiety, but these are subjugated to the gastrointestinal ones. There are now a number of specific and effective treatments for IBS that either ignore the generalised anxiety components or describe them in terms that are specific to the organ system concerned.

### Muscular pain and tension – fibromyalgia

Fibromyalgia is a complex word, but pain and tension are the most prominent symptoms. There are many who doubt the value of this diagnosis, but few can deny that it has taken two important symptoms away from GAD.

### Other bodily symptoms – somatic symptom and somatoform disorders

There are many other bodily symptoms that have been given formal titles in general medicine – non-cardiac chest pain, respiratory distress syndrome, functional dysphagia – but they all are selective in picking out one of the symptoms of GAD and ignoring the others. Somatoform disorders are ripe for justified criticism,^2^ but they are overshadowed by a proto-diagnosis that is not liked for its aetiological nihilism, ‘medically unexplained symptoms’; it gets a great deal of interest and attention, especially in primary care, again taking a chunk away from the GAD superstructure.

## Worry about health matters

People with the symptoms of GAD whose main symptom is worry about their health are now diagnosed with illness anxiety (in the USA) or health anxiety (outside the USA), but as these describe the same condition we should not get too concerned. In health anxiety, minor worries about health are amplified into a full anxiety syndrome that includes all the symptoms of GAD. One of the core parts of treatment is for the patient to learn to appreciate that these ‘minor matters’ should remain minor, so chipping away at another GAD limb.^3^

## Recurrent disorder that may be lifelong

Anxiety is a personality trait as well as a symptom, and it has been appreciated for many years that anxious or avoidant personality features are very common in people with GAD. ‘I've always been a worrier’ and ‘I come from an anxious family’ are very common spontaneous comments made by patients at assessment. So, when patients with GAD construct a life chart of their anxiety, it is easy to identify a range of outcomes from one episode only to dozens of clear-cut episodes over time, with many others that may not reach the threshold for GAD. The patient often does not make much of this diagnostic distinction, as the handicap created by the symptoms at different times is very similar. But it makes prognosis extremely difficult to determine.

There is so much overlap between personality status and GAD that a strong case can be made for a condition called the ‘general neurotic syndrome’ (or the ‘general nervous syndrome’ for those who abhor the word ‘neurotic’), in which certain personality features such as obsessionality and dependence, as well as anxiousness, contribute to the biaxial diagnosis.^4^

## Mixed anxiety–depression (cothymia)

‘The study of anxiety and depression

Teaches us one important lesson

Though their separate study often pleases

We must remember they are not diseases

Like wind and rain in stormy weather

These symptoms always come together’^5^

This is the real cruncher that fells GAD completely. Nosologists have tried desperately for years to keep anxiety and depression apart, insisting that they are distant relatives, not siblings, but the family bonds are too strong and the two always hover together threateningly at diagnostic gatherings. The analogy is appropriate, as the genetic evidence has shown repeatedly that anxiety and depression have a common genetic structure^6^ and so should be thought of together, despite the obvious differences in symptomatology.

Despite the valiant efforts of David Goldberg^7^ and several others, there has been great reluctance to accept mixed anxiety–depression as a full syndromal diagnosis in either the DSM or ICD classifications. If this was agreed, and it is becoming increasingly likely this will happen, the separate diagnosis of GAD would be weakened severely.

## Unreliability of diagnosis

Some of these criticisms could be overcome if clinicians were pleased with the criteria for the diagnosis and used them consistently. But they do not. In the recent field trials of DSM-5, the interrater reliability of GAD was 0.20,^8^ which the authors regard as ‘questionable’, but which most clinicians would regard as unacceptable.

## Replacement of GAD

If we abandoned GAD, what would replace it? There are three possibilities here. The first is to elevate mixed anxiety–depression to a more robust diagnosis instead of an apologetic afterthought. But of course, some would argue correctly that generalised anxiety can occur in the absence of depression. The second is to be really bold and join up the common personality characteristics of anxiety, dependence and obsessionality with the mood disturbance to constitute a ‘general neurotic syndrome’. This is a condition, usually combined with depression, that runs a chronic course and has a worse long-term outcome than either anxiety or depressive disorder alone,^4^ especially when the personality disorder is more severe.^9^ The third option is to think of GAD as an adjustment disorder.

## Adjustment disorder

Many people receive a diagnosis of GAD when they are somewhat anxiety prone and then experience a major life event, especially one that is perceived as threatening. Although for many years adjustment disorder had been thought of as a subsyndromal diagnosis, and as a consequence largely ignored,^10^ it is now being examined more seriously as an important and measurable element of the trauma-focused disorders.^11^ The importance of this in clinical practice is that that these life event-precipitated forms of GAD could be treated by relatively brief psychological therapies and be less likely to lead to long-term iatrogenic disease.

## Conclusions

The old concept of ‘anxiety neurosis’ has gone and been replaced by a host of different labels. In the course of this process, the original core of free-floating anxiety coming from out of the blue and surrounding each patient with a mist of uncertainty and threat has become redundant. It is persistently comorbid with other conditions and has no central elements that deserve separate classification. It should be quietly laid to rest and little mourned.

## About the author

**Peter Tyrer** is Emeritus Professor of Community Psychiatry, Centre for Psychiatry, Imperial College, London, UK.

## References

[1] Grzesiak M, Beszłej JA, Mulak A, Szechiński M, Szewczuk-Bogusławska M, Waszczuk E, The lifetime prevalence of anxiety disorders among patients with irritable bowel syndrome. Adv Clin Exp Med 2014; 23: 987–92. 25618127 10.17219/acem/37356

[2] Mayou R. Is the DSM-5 chapter on somatic symptom disorder any better than DSM-IV somatoform disorder? Br J Psychiatry 2014; 204: 418–9. 25029686 10.1192/bjp.bp.113.134833

[3] Tyrer P, Eilenberg T, Fink P, Hedman E, Tyrer H. Health anxiety: the silent treatable epidemic. BMJ 2016; 353: i2250. 27112356 10.1136/bmj.i2250

[4] Tyrer P, Tyrer H, Guo B. The general neurotic syndrome: a re-evaluation. Psychother Psychosom 2016; 85: 193–7. 27230860 10.1159/000444196

[5] Tyrer P. Anxiety: a multidisciplinary review. Imperial College Press, London, 1999.

[6] Hettema JM, Aggen SH, Kubarych TS, Neale MC, Kendler KS. Identification and validation of mixed anxiety-depression. Psychol Med 2015; 45: 3075–84. 26050714 10.1017/S0033291715001038

[7] Goldberg DP. Anxious forms of depression. Depress Anxiety 2014; 31: 344–51. 24281827 10.1002/da.22206

[8] Regier DA, Narrow WE, Clarke DE, Kraemer HC, Kuramoto SJ, Kuhl EA, DSM-5 field trials in the United States and Canada, Part II: test-retest reliability of selected categorical diagnoses. Am J Psychiatry 2013; 170: 59–70. 23111466 10.1176/appi.ajp.2012.12070999

[9] Tyrer P, Tyrer H, Yang M, Guo B. Long-term impact of temporary and persistent personality disorder on anxiety and depressive disorders. Personal Mental Health 2016; 10: 76–83. 10.1002/pmh.1324 26754031

[10] Casey P. Adjustment disorder: new developments. Curr Psychiatry Rep 2014; 16: 451. 24748555 10.1007/s11920-014-0451-2

[11] Bachem R, Perkonigg A, Stein DJ, Maercker A. Measuring the ICD-11 adjustment disorder concept: validity and sensitivity to change of the adjustment disorder - new module questionnaire in a clinical intervention study. Int J Methods Psychiatr Res 2016; doi: 10.1002/mpr.1545. PMC6877162 27862575

